# Newly synthesized chitosan nanoparticles loaded with caffeine/moringa leaf extracts Halt Her2, BRCA1, and BRCA2 expressions

**DOI:** 10.1038/s41598-024-67599-1

**Published:** 2024-08-05

**Authors:** Hanaa Mohammed, Mustafa M. Karhib, Karrar Sabah Jaafar Al-Fahad, Atef Mohamed Atef, Areej Eskandrani, Amira Abd-elfattah Darwish, Ahmed Abdallah Sary, Bassma H. Elwakil, Basant A. Bakr, Ahmed M. Eldrieny

**Affiliations:** 1 Human Anatomy and Embryology Department, Faculty of Medicine, Sohag University, Sohag, Egypt; 2 Department of Medical Laboratory Techniques, College of Health and Medical Technologies, Al-Mustaqbal University, Hillah, Babylon 51001 Iraq; 3 Babylon Education Directorate, Ministry of Education, Hillah, Iraq; 4 Faculty of Medical Applied Science, Irbid National University, Irbid, Jordan; 5 College of Science, Taibah University, 30002 Madinah, Kingdom of Saudi Arabia; 6 Faculty of Applied Health Sciences Technology, Pharos University in Alexandria, Alexandria, 21526 Egypt; 7 Faculty of Physical Therapy, Pharos University in Alexandria, Alexandria, 21526 Egypt; 8 Faculty of Science, Alexandria University, Alexandria, 21321 Egypt

**Keywords:** Breast cancer, Natural anticancer agents, Nano carrier, Chitosan, MCF-7, Her2, BRCA1, BRCA2, mTOR, Breast cancer, Cancer therapy, Nanoscience and technology

## Abstract

Breast cancer is among the highest morbidity and mortality rates in women around the world. In the present investigation we aimed to synthesis novel nanosystem combining two naturally important anticancer agents with different mechanism of action namely *Moringa oleifera* and caffeine. Firstly, chemical analysis of *Moringa oleifera* extract and caffeine was done by gas chromatography-mass spectroscopy (GC–MS) in order to assess the main chemical compounds present and correlate between them and the possible anticancer effect. The novel nanosystem was characterized through dynamic light scattering techniques which revealed the stability and homogeneity of the prepared *M. oleifera* leaves extract/Caffeine loaded chitosan nanoparticles, while FTIR and transmission electron microscope (TEM) proved the shape and the successful incorporation of *M. oleifera* leaves extract/Caffeine onto the nanochitosan carrier. Our initial step was to assess the anticancer effect in vitro in cancer cell line MCF-7 which proved the significant enhanced effect of *M. oleifera* leaves extract/Caffeine nanosystem compared to *M. oleifera* leaves extract or caffeine loaded nanoparticles. Further studies were conducted in vivo namely tumor biomarkers, tumor volume, bioluminescence imaging, molecular and histopathological investigations. The present study proved the potent anticancer effect of the synthesized *M. oleifera* leaves extract/Caffeine loaded chitosan nanoparticles. Mo/Caf/CsNPs exhibited a large number of apoptotic cells within the tumor mass while the adipose tissue regeneration was higher compared to the positive control. The prepared nanoparticles downregulated the expression of Her2, BRCA1 and BRCA2 while mTOR expression was upregulated. The aforementioned data demonstrated the successful synergistic impact of Moringa and caffeine in decreasing the carcinoma grade.

## Introduction

Breast cancer (BC) has the highest cancer mortality rates among women worldwide^1^. The global BC statistics report shows that in 2020, 2.261 million new cases and 685,000 deaths worldwide were reported. It was estimated that by 2050, the age standardized rate of BC incidence in female will be 59.63 per 100,000, an increase of 32.13% compared with 2019^2^. BC progression is usually verified by the decline in the estrogen receptor-a (ER) which implies an additionally aggressive tumor with endocrine therapies (e.g. tamoxifen) failure^3^. ER can cause cellular proliferation through progesterone receptor (PgR) activation and transcriptional increment of cyclin D1, resulting in improved cell-cycle progression, while ER declining has been linked to increased movements and metastatic ability of BC cells. Changes in ER levels could be controlled at the transcriptional or posttranscriptional levels, as well as through epigenetic processes^4^. BC’s frequency differs by geography, potentially modifiable environmental, nutritional, and other lifestyle factors which may impact the ER status also. The insulin-like growth factor (IGF) family was discovered as another risk factor for breast cancer. The downstream of IGF type I receptor (IGFIR) targets MAPK-ERK or PI3K-Akt that finally mediates the mitogenic and antiapoptotic signals. While, ER and IGFIR signaling pathways are inextricably coupled and susceptible to feedback crosstalk. Estrogen and IGFs co-regulate numerous genes and usually influence the fate of breast cancer progression^5^. Furthermore, hyperactive IGFIR-PI3K-Akt signaling has been linked to the development of endocrine treatment’s resistance during BC progression^6^. On the other hand, Human epidermal growth factor receptor type 2 (HER-2) is an important molecular biomarker in BC (over expression 20- to 30-times during primary malignancy)^7^. Hence, ER and HER-2 genes are significantly important predictor genes in breast cancer therapy effectiveness^7^. Heat shock proteins (HSPs) are abundant in the mitochondria and play an important role in cancer development^8^. Specifically, Hsp90 interacts with other proteins involved in breast neoplasia, such as estrogen receptors (ER), the tumor suppressor protein p53, the antiapoptotic kinase Akt, the Raf-1 MAP kinase, and various receptor tyrosine kinases, such as HER-2^9^. Hence, tissue-based and serum-based circulating biomarkers are significant in BC management. CA 15.3, CA 27.29 and MMP 9 are the most thoroughly studied serum tumor markers in BC^10^. CA 15.3 and CA 27.29 are soluble versions of the transmembrane glycoprotein MUC-1 (mucin protein)^11^. MUC-1 is found on the apical cell surface of epithelia of the respiratory tract, GI tract, female reproductive system, and mammary gland^12^. As a result, in physiological conditions MUC-1 helps with cell proliferation, survival, and epithelial integrity. Unfortunately, MUC-1 is overexpressed in many forms of carcinomas, where cancerous cells seize MUC-1 signal transduction pathways to boost their proliferation, invasiveness, and metastatic capacity^13^.

It was noticed that adjuvant treatments for breast cancer have adverse effects along with systemic toxicity and some cancer cells may develop resistance^14^. These limitations have made it necessary to look for more effective and reasonably priced cancer treatments. In this context, medicinal herbs represent a significant alternative strategy since they have fewer side effects. In African communities, the *Moringa oleifera* Lam. (Mo) leaf is a popular herbal remedy for the treatment of cancers and called the “Miracle Tree”^15^. Its effectiveness against breast cancer has been established in several in vitro investigations^16,17^. Several experimental research have been conducted over the last decade to investigate the phytochemical composition of Moringa leaves, with an emphasis on the key role of its antioxidant activity. Previous research has extensively documented the leaves' pharmacological properties^18^. The considerable presence of ascorbic acid, vitamins A, B, α -tocopherol, β-carotene, β-sitosterol, protein, and specifically essential amino acids detected in Moringa leaves and pods are characteristic components that made Moringa an ideal nutraceutical^19^.

On the other hand, scarce translational research on the anticancer benefits of coffee in relation to ER status in breast cancer has been conducted. Coffee drinking is associated with a considerably lower risk, delayed onset, and decreased development of BC, according to prospective and retrospective epidemiologic research^20^. Caffeine (major component in the coffee beans) has high antioxidant effect and hence it may be implicated in coffee's cancer-fighting qualities^21^. In accordance with this, a subgroup of cohort study previously demonstrated that moderate to high coffee intake was correlated with the increased disease-free survival among ER tamoxifen-treated women^22^.

However, the underlying issues of some naturally derived bioactive chemicals are their insolubility and fast denaturalization in the physiological milieu has been extensively addressed^23^. Nanomedicine is a field of study that entails comprehensive monitoring, repair, control, defense, construction, and molecular improvement of biological systems using engineered nanostructures to accomplish higher health-benefits. The development of cancer nanomedicine has focused on increasing and improving the anticancer agents’ efficacy and delivery into tumor cells^24^.

**Figure 1:**
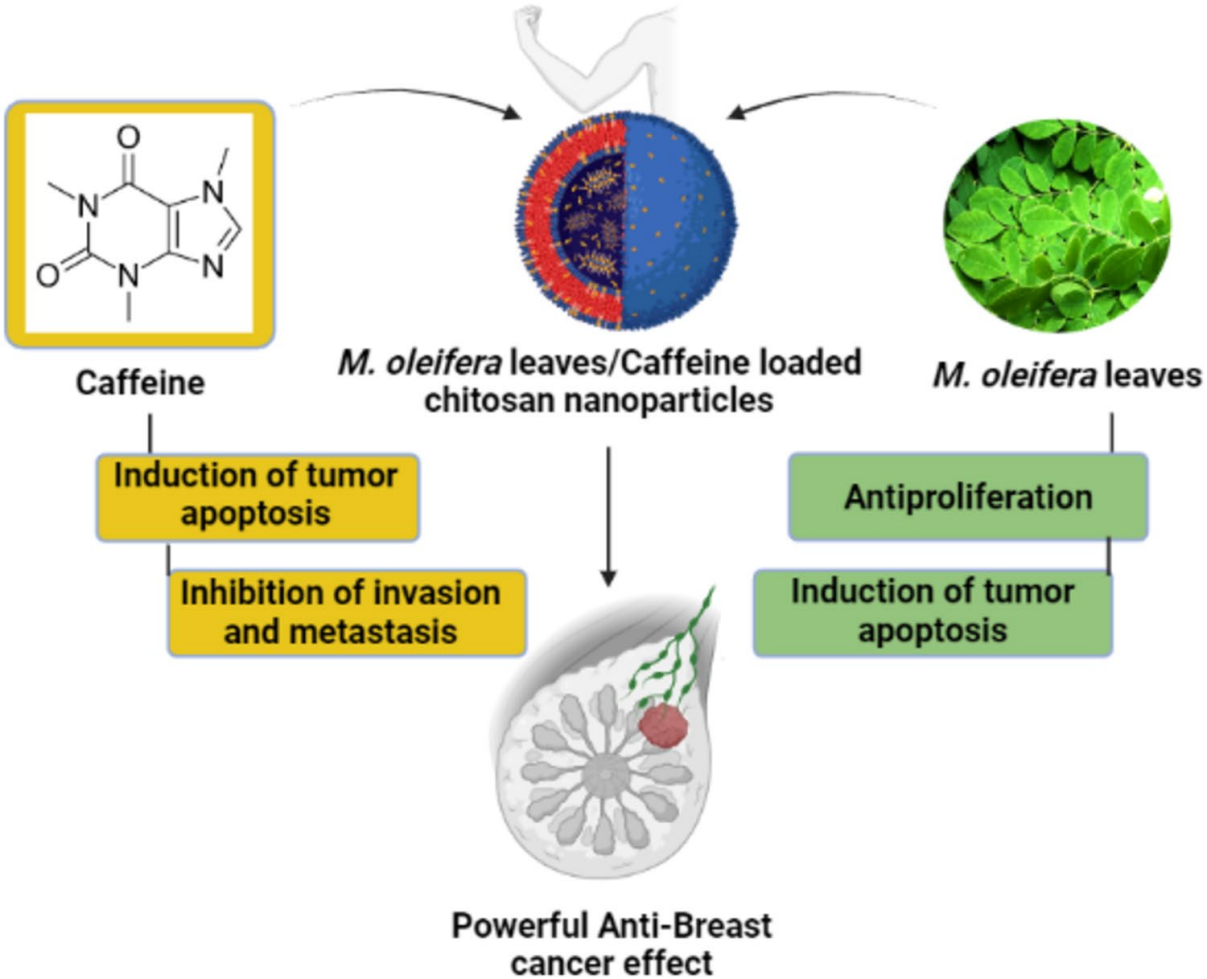
Study rational.

Hence, the aim of the present work was to synthesize novel nanoplatform composed of Moringa leaves extract and caffeine to synergistically combat breast cancer in vitro and in vivo (Fig. 1). As it was previously reported that *M. oleifera* leaves extracts have antiproliferation effect while caffeine can induce tumor apoptosis hence combining both these natural products can battle BC by dual acting agent.

## Materials and methods

### Materials

CA 15.3, CA 27.29 and MMP 9 Enzyme Immunoassay kit were purchased from MYBIOSOURCE (San Diego, CA, USA). Caffeine (C0750) was purchased from Sigma-Aldrich (MO, USA). Human breast adenocarcinoma cell line (MCF-7) and Mouse mammary adenocarcinoma 4T1 cells were provided from ATCC (American Type Culture Collection, Manassas, VA, USA). The fully developed *Moringa oleifera* leaves were gathered from the native store during Fall 2023 in Siwa governorate (29.2032° N, 25.5195° E), Egypt. The gathering of *Moringa oleifera* leaves complied with relevant institutional, national, and international guidelines and legislation.

## Microwave assisted *Moringa* oleifera leaves extraction

A microwave oven (900 W, 50 Hz, TM-25MS) was used for *Moringa oleifera* extraction. *M. oleifera* leaves (50 g) were washed, cut, grided and macerated in deionized distilled water (1000 mL). The mixtures were irradiated at 750 W, for 90 s as described by Wanjiru et al.^25^. The crude extract was filtered, freeze-dried and stored at − 20 °C.

### Chemical analysis

GC–MS analysis was used to assess the chemical structure of the extracted *M. oleifera* leaves and the solubilized caffeine powder^26^. The instrument had an injection port and detector temperature 275–300 °C, column temperature 180 °C, flow rate 35 mL/min with flame-ionization detector.

### Nanoparticles synthesis and characterization

#### *Moringa oleifera* leaves extract or Caffeine loaded CsNPs preparation (Mo/CsNPs and Caf/CsNPs)

Caffeine or *M. oleifera* leaves extract loaded chitosan nanoparticles (CsNPs) were prepared through ionic gelation method according to Aljohani et al.^27^ with some modifications. Cs solution was prepared (1% w/v) in acidified water (1% acetic acid v/v) while TPP solution was freshly prepared (0.2% w/v in deionized water). Eight mL of filtered Caffeine solution or *M. oleifera* leaves extract (400 mg) one at a time were mixed thoroughly with the TPP solution. The mixture was then poured drop by drop to the freshly produced Cs solution and stirred for 15 min.

#### *Moringa oleifera* leaves extract/Caffeine loaded CsNPs preparation (Mo/Caf/CsNPs)

The preparation of caffeine/*M. oleifera* leaves extract loaded chitosan nanoparticles was a novel formulation. Cs and TPP solutions were made as formerly stated. Four ml of each solution (Caffeine solution or *M. oleifera* leaves extract solution) (400 mg each) were mixed for 10 min under stirring condition then the mixture was added to the freshly TPP solution. The mixture was then poured drop by drop to the freshly produced Cs solution and stirred for 15 min.

### Nanoparticles characterization

The size and shape of the produced NPs were assessed using a transmission electron microscope (TEM, JEOL, JSM-2100 Plus, Japan). The NPs' zeta potential, zeta size and PDI were calculated using the dynamic light scattering (DLS) method through Malvern Zetasizer. FTIR, and entrapment efficiency (EE%) studies were assessed according to Aljohani et al.^27^.

### In vitro study

Human breast adenocarcinoma cell line (MCF-7) (ATCC, Manassas, VA, USA) was maintained at 5% CO_2_, 37 °C in DMEM. MTT assay was used to determine the cell viability of MCF-7 cells (1 × 10^4^ cell/well). After cells attachment, different concentrations of Mo/CsNPs, Caf/CsNPs and Mo/Caf/CsNPs were incubated for 24 h and 48 h according to Talaat et al.^28^.

### In vivo assessments

**Figure 2:**
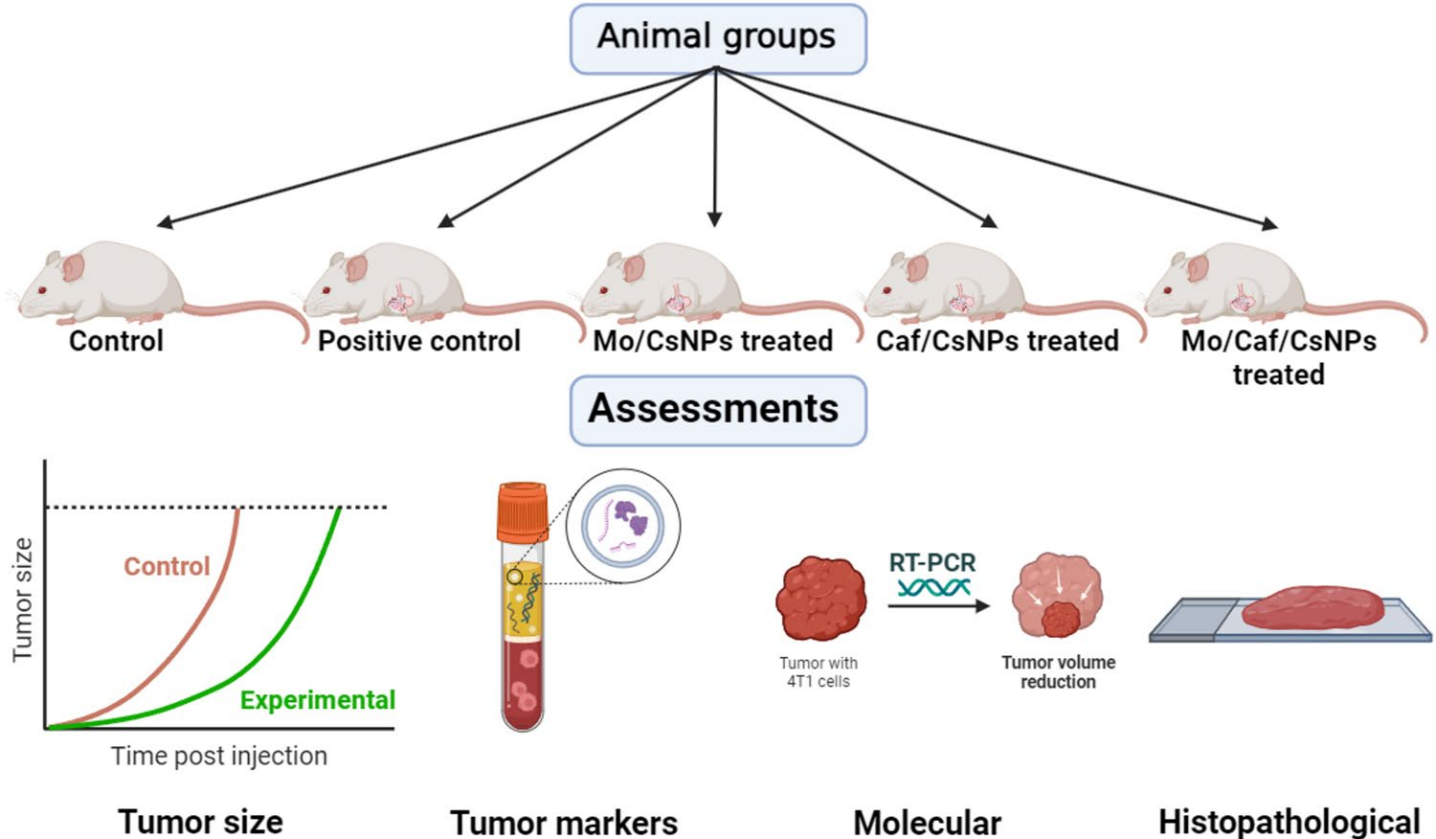
Animal grouping and assessments.

BALB/c female mice (5 weeks old) were used in the experiments. Animals were grouped into 5 groups each group including 5 mice and kept in a cage under conventional conditions of temperature (25 ± 2 °C) and humidity. During the experiment, mice had access to drinking water ad libitum. All procedures were performed in accordance with the guidelines of the ethical committee of Pharos University in Alexandria, Egypt (Approval No.: PUA/04/2024/02/25/3/204) and comply with ARRIVE guidelines and the National Institute of Health for the care and use of laboratory animals. Mouse mammary adenocarcinoma 4T1 cells (1 × 10^5^ cells/mouse) were inoculated subcutaneously into the abdominal mammary gland area (syngeneic model)^29^. After the tumor mass was visible, the treatments regimens started (oral administration daily for 15 weeks (the end of the study))^30^. Mice were divided into (n = 5) (Fig. 2):

1. Control: Normal mice (received saline, oral administration)
2. Positive control (PC): Breast cancer without treatment
3. *M. oleifera* leaves extract loaded CSNPs (Mo/CsNPs): Breast cancer mice treated with *M. oleifera* leaves extract loaded CSNPs (100 mg/kg, orally).
4. Caffeine loaded CsNPs (Caf/CsNPs): Breast cancer treated with caffeine loaded CSNPs (100 mg/kg, orally).
5. *M. oleifera* leaves extract/Caffeine loaded CsNPs (Mo/Caf/CsNPs): Breast cancer treated with caffeine/*M. oleifera* leaves extract loaded CSNPs (100 mg/kg, orally).

After 15 days, each mouse was intraperitonially injected with ketamine (45 mg⁄kg) and xylazine (35 mg⁄kg) mixture for anesthetization. Blood samples were collected while tumors tissues were dissected and rinsed in ice-cold 1.15% KCl, dried on filter paper, weighed and photographed. Digital images were processed using ImageJ software to assess the major and minor axes of the tumor and determine the tumor length (mm). The percentage change in tumor volume relative to baseline volume was used to estimate tumor growth inhibition. Tumor volume was assessed using the following equation (Eq. 1)^28^:

1 $$\text{Tumor volume}=\text{ length }\times {(\text{width})}^{2}\times 0.5$$

### Determination of tumor marker

Serum CA 15.3, CA 27.29 and MMP 9 concentrations were determined using an Enzyme Immunoassay kit based on Sandwich ELISA according to the manufacturer’s instructions.

### Histopathological analysis

Breast tumors were cut into pieces, which were fixed in 4% paraformaldehyde for 24 h before being embedded in paraffin. The specimens were sectioned at 4 μm thickness and stained with hematoxylin–eosin according to the routine processing protocol of Bancroft and Gamble^31^. Tumor grade and treatment efficacy were assessed by histopathological examination of sections taken from the different experimental groups using a light microscope equipped with a camera.

### Immunohistochemical analysis

Tissue sections (4 μm thick) were fixed on poly-l-lysine-coated slides, deparaffinized in xylene, and then rehydrated using ethanol series. Epitope retrieval was completed by samples’ heating in the microwave for 3 min in a 10 mM sodium citrate (pH 6.0) solution. After serial washing then blocking in a 5% bovine serum albumin, samples were incubated with Ki-67 antibody (1:500, Cell Signaling Technology, catalog no. 12202S). Using the image analysis program Image J, two independent observers manually counted the numbers of Ki-67 positive cells in five randomly chosen fields.

### Real time quantitative PCR

Homogenized tumour tissues were weighed (50 mg) then subjected to RNA extraction kit (Easy red total RNA extraction kit, Intronbio, Korea) according to manufacturer’s instructions. After RNA (1 μg) extraction and purification, cDNAs were synthesized (PrimeScript RT Master Mix, TAKARA, Kyoto, Japan). HER-2, two tumour suppressor genes; breast cancer gene 1 (BRCA1), and breast cancer gene 2 (BRCA 2), and cell angiogenesis gene mTOR (all primers were purchased from willowfort.co.uk) were tested in the present study (Table 1). Values from β-actin was used to loading normalization for each sample. Relative changes expression was determined using the 2^− ΔΔ Ct^ method^28–33^.

**Table 1:** Sequence of specific primers used for quantitative real-time revers transcription PCR.

Her2-F	GGCTTGGTCTGTAACTCACTG
Her2-R	TTCCATACTCGGCACTCCTC
BRCA1-F	TGAAGACTGCTCGCAGAGTGATA
BRCA1-R	AGCTTCCAGGTGAGCCATTTC
BRCA2-F	TTGAGGACCCCAAGACCTGT
BRCA2-R	CCGGAGAGACAAAGGTGCA
mTOR-F	GCTTGATTTGGTTCCCAGGACAGT
mTOR-R	GCTTGATTTGGTTCCCAGGACAGT
β-actin-F	TGAAGATCAAGATCATTGCTCCTC
β-actin-R	TCAGTAACAGTCCGCCTAGAAG

### Bioluminescence imaging

Animals were rested in a CT (computerized tomography) and PET (positron emission tomography) after 30 min of the administration of 0.2 mCi 18F-FDG. A CT scan (X-ray energy, 40 kV; intensity, 140 A) was carried out after each PET capture to allow for subsequent attenuation correction in image reconstruction and unambiguous radioactive signal localization according to Aljohani et al.^27^.

### ‏ Statistical analysis

Student’s *t*-test (*p* ≤ 0.05) was used to statistically analyse our results. Comparison between the studied groups was carried out using F-test (ANOVA) and post hoc test (Tukey) for pair-wise comparisons (GraphPad Prism version 6). All statistical analyses were two-sided and the significance of the acquired results was determined at the 5% level^34^.

### Institutional review board statement

This research work was approved for publication by unit of research ethics approval committee (UREAC), Faculty of Pharmacy, Pharos University in Alexandria (PUA/04/2024/02/25/3/204).

## Results

### GC–MS analysis

**Figure 3:**
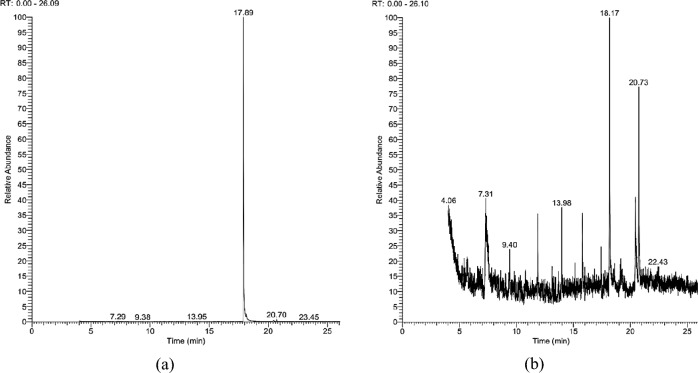
GC–MS analyses of caffeine (**a**) and *Moringa oleifera* leaves extract (**b**).

Chemical analysis of purchased caffeine proved the compound purity (Fig. 3a). *M. oleifera* leaves extract’s chemical analysis (Fig. 3b) showed some potent compounds with various biological properties (Table 2). It was revealed that Octane, 3,5-dimethyl, Methyl 12-methyltetradecanoate, and Methyl 15-methylhexadecanoate (19.86, 17.89 and 11.99 area % respectively) were the most abundant compounds.

**Table 2:** Chemical analysis of *M. oleifera* leaves extract.

RT	Area %	Compound name	Uses
6.67	1.17	Lycoxanthin	Antimicrobial, anti-inflammatory and anticancer^35^
7.31	19.68	Octane, 3,5-dimethyl	Allelochemical effect^36,37^
9.40	3.21	Astaxanthin	Antioxidant, anti-Lipid peroxidation, anti-diabetic, anti-inflammatory, and anticancer^38^
10.81	1.63	2,4-Imidazolidinedione, 5-[3,4-bis[(trimethylsilyl)oxy]phenyl]-3-methyl-5-phenyl-1-(trimethylsilyl)-	Anti-lipid peroxidation, cyclooxygenase inhibition activities^39^, Anticancer activity^40^
13.14	1.48	4H-Pyran-4-one, 2,3-dihydro-3,5-dihydroxy-6-methyl-	Antioxidant^41^, Protective effect on male reproductive functions^42^
13.98	4.39	2-Propenoic acid, 3-(3,4,5-trimethoxyphenyl)-, methyl ester	Anti-inflammatory^43^
16.85	1.15	9,19-Cyclolanostan-24-one, 3-acetoxy-25-methoxy	Anti-inflammatory^44^
18.7	17.89	Methyl 12-methyltetradecanoate	Wound healing probability^45^
20.73	11.99	Methyl 15-methylhexadecanoate	Food preparation, baking, cosmetics and pharmaceutics^46^

### Nanoparticles characterizations

**Figure 4:**
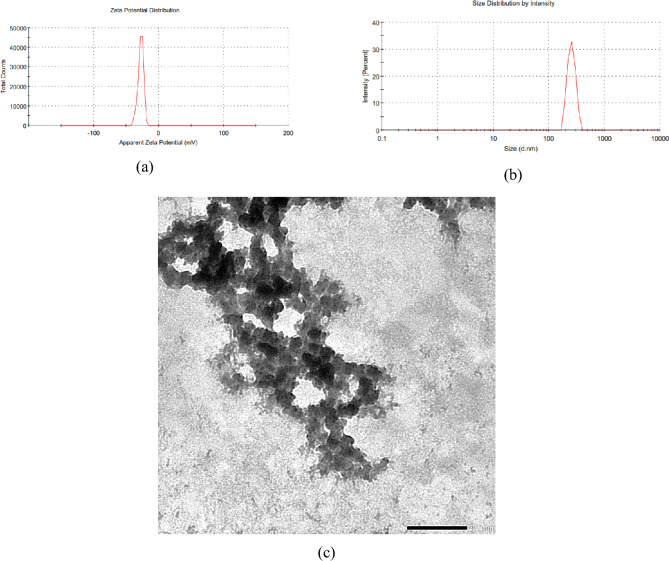
DLS (**a**,**b**) and TEM (at scale bar 200 nm) studies (**c**) of Mo/Caf/CsNPs.

**Figure 5:**
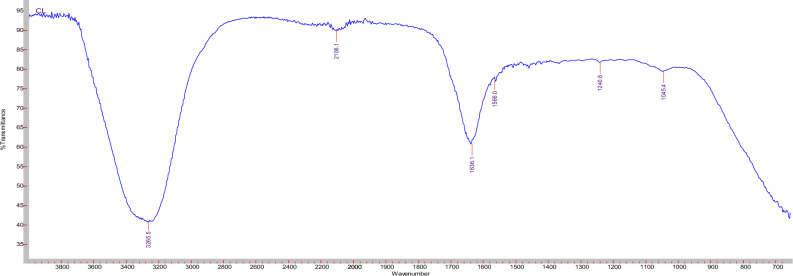
FTIR analysis of the prepared Mo/Caf/CsNPs.

TEM study of the prepared nanosystems revealed that Mo/Caf loaded chitosan nanoparticles had an almost rectangular shape (Fig. 4). Further analyses showed that the combination between Moringa leaves extract and caffeine solution enhanced the physicochemical properties of the prepared nanosystem (Mo/Caf/CsNPs) **(**Table 3**)**. FTIR analysis showed several intense bands in the wave number region between 4000 and 400 cm^−1^. Strong peak present at 3252 cm^−1^ in Mo/Caf/CsNPs resemble OH stretching (Fig. 5 and Fig. S1). Overall, the FTIR results show the successful nanoparticulate system composed of Mo/Caf/CsNPs.

**Table 3:** Physicochemical parameters of the prepared nanosystems.

Nanosystems	Zeta size (nm)	Nanoparticles diameter (nm)	Zeta potential (mV)	PDI	EE%
Mo/CsNPs	379	58.2	− 40.7	0.285	70.0
Caf/CsNPs	248	64.1	− 31.3	0.320	62.4
Mo/Caf/CsNPs	369	70.4	− 36.8	0.321	81.0

### In vitro study

**Figure 6:**
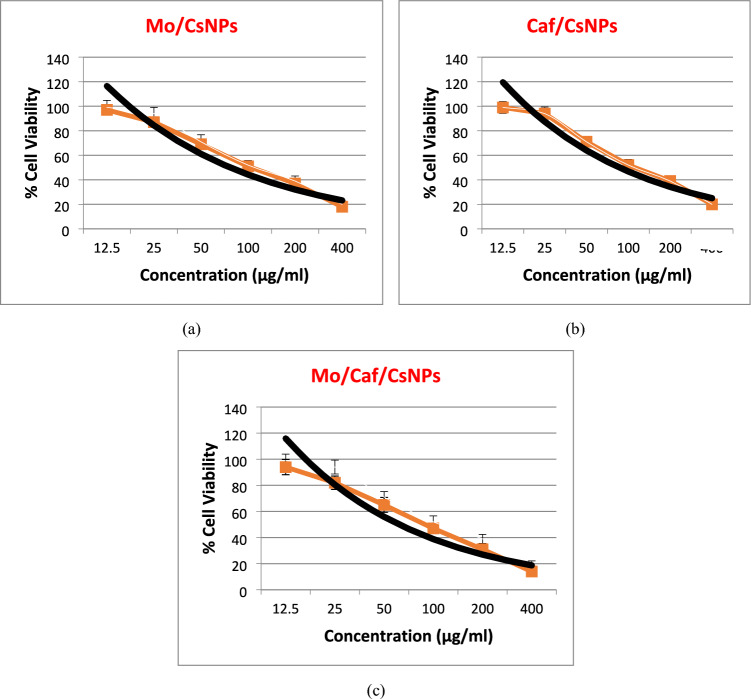
Cytotxic effect of Mo/CsNPs (**a**), Caf/CsNPs (**b**) and Mo/Caf/CsNPs (**c**).

The cytotoxicity of Mo/CsNPs, Caf/CsNPs and Mo/Caf/CsNPs were tested at different concentrations on MCF-7 (human breast cancer cells), using MTT assay. Figure 6 showed the cytotoxicity results of Mo/CsNPs, Caf/CsNPs and Mo/Caf/CsNPs after incubation with MCF-7 cells for 24 h. As shown in Fig. 6, both Mo/CsNPs and Caf/CsNPs demonstrated significant reduction in cell viability in a time- and dose-dependent manner. However, at the same concentration, Mo/Caf/CsNPs showed significantly (*P* < 0.05) high proliferation inhibitory effect which was expressed as lower IC_50_ values (Table 4).

**Table 4:** IC_50_ of the tested nanosystems.

Formula	IC_50_ (24 h)	IC_50_ (48 h)
Mo/CsNPs	110.12	55.09
Caf/CsNPs	100.63	50.56
Mo/Caf/CsNPs	76.04	37.81

### In vivo study

#### Tumor biomarkers

**Figure 7:**
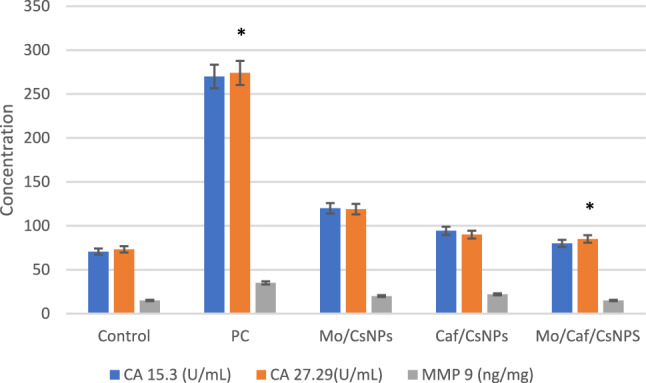
Immunological biomarkers levels among the study groups. *P < 0.001 from positive control (data were described using mean and standard deviation for normally distributed data).

According to the presented data in Fig. 7, using mix of moringa leaves and caffeine has powerful effect on breast cancer biomarkers. Data reveled that Mo/Caf/CsNPs had a significantly (*p* < 0.05) different effect on CA 15.3, CA 27.29 and MMP 9 expression when compared to the positive control group. It can be exploited to generate novel medications for the treatment of breast tumors efficacy.

### Bioluminescence study

**Figure 8:**
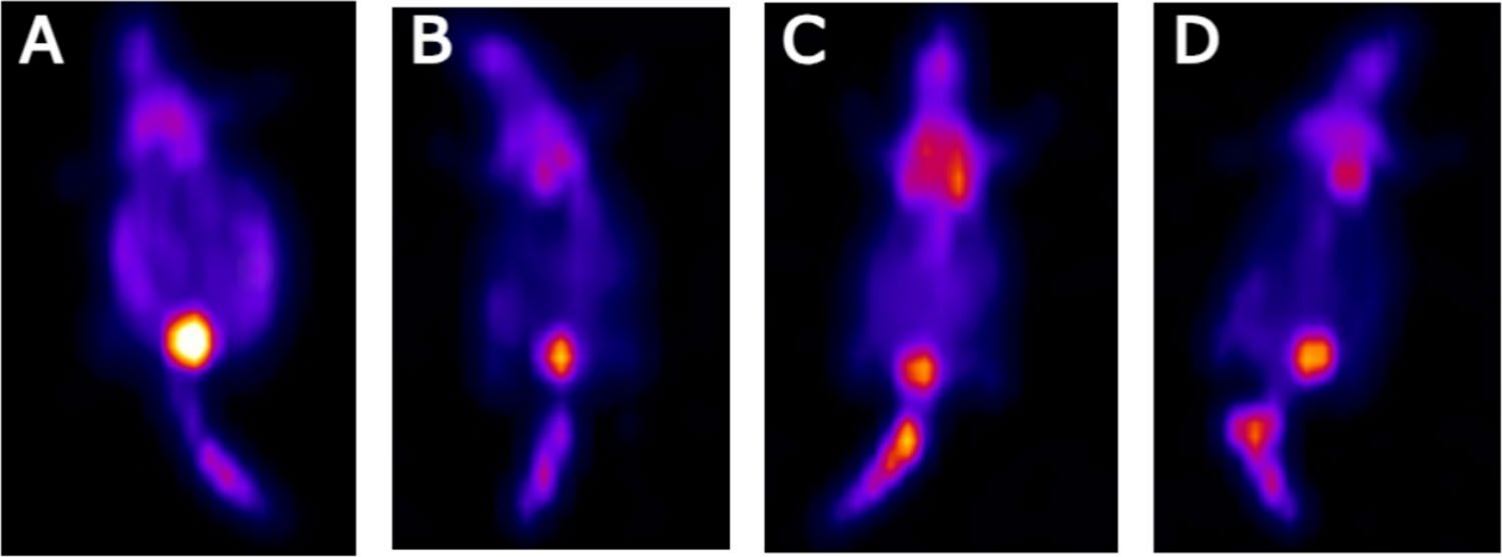
Bioluminescence imaging of positive control (**A**), Mo/CsNPs (**B**), Caf/CsNPs (**C**) and Mo/Caf/CsNPs (**D**) groups.

Breast tumors (positive control) showed a strong bioluminescent signal (Fig. 8). The signal was weaker in the other tested groups’ bioluminescence imaging.

### Histopathological evaluation

Breast cancer is characterized as either ductal or lobular based on its shape and likely location of origin. Through reviewing three tumor criteria, all breast tumors may be histologically graded using the Nottingham Grading System (NGS), commonly known as the Elston-Ellis grading system. In this system, the parameters examined are (i) the degree of tubular differentiation, (ii) nuclear pleomorphism, and (iii) mitotic activity graded, that high grade linked with poor overall survival^47^. Herein, the Breast Cancer Ehrlich model was used to analyze the efficiency of various natural compounds as anti-cancer therapy, as well as to evaluate their apoptotic and necrotic activity.

**Figure 9:**
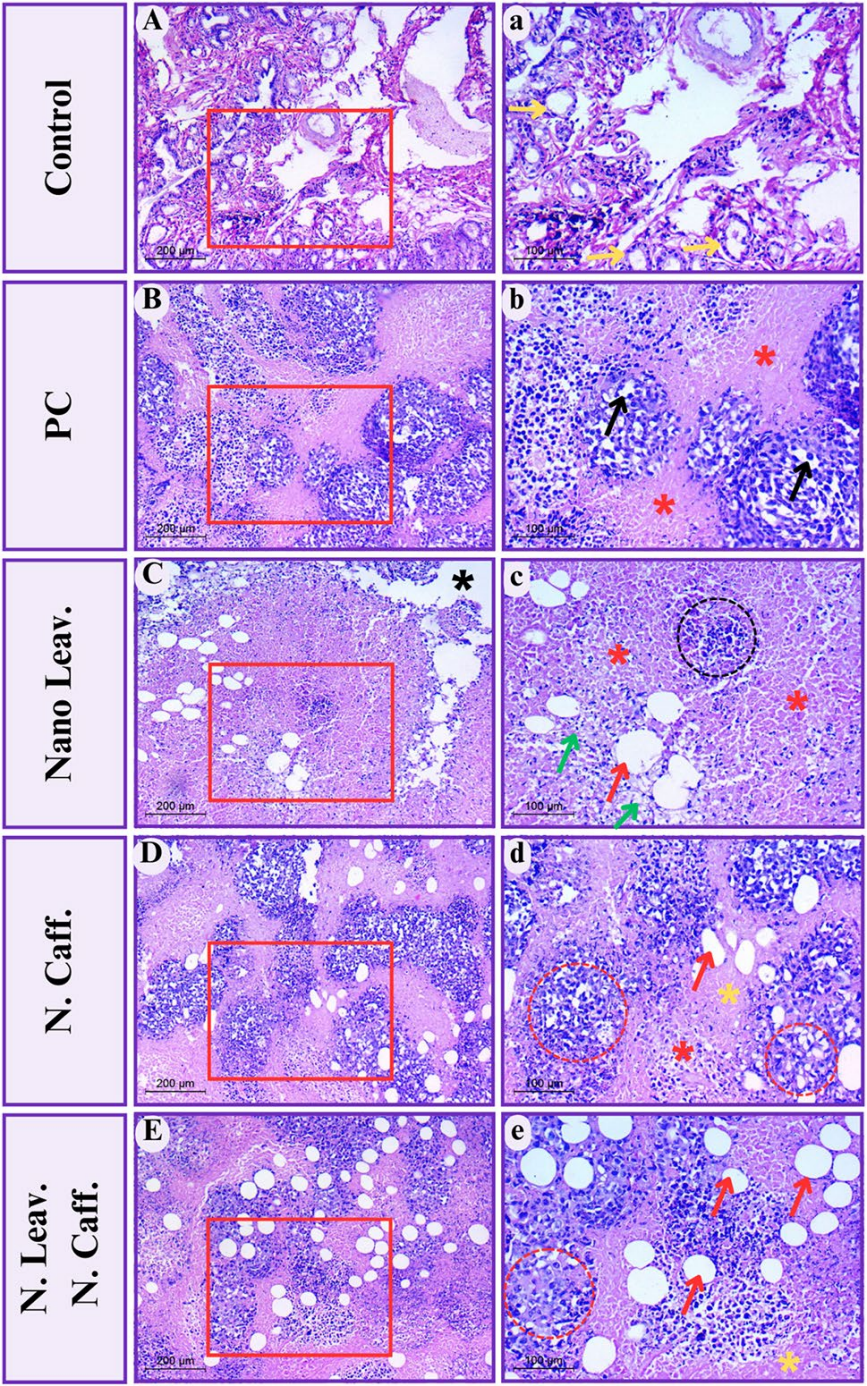
Photomicrographs illustrate several H&E-stained tissue specimens of mice breast (magnification × 400, (inset) ×200), (**A-a**) normal control breast showing typical TEBs (yellow arrows), (**B-b**) positive control group (PC) showing Plasmacytoid cells (black arrows), necrotic areas (red asterisks); (**C-c**) N. Leav group showing necrotic areas (red asterisks), tumor cell infiltrations (black circle), fibrosis (green arrows) and little regeneration of adipose tissue (red arrow); (**D-d**) N. Caff group showing apoptotic areas (yellow asterisks), necrotic areas (red asterisks), damaged tumor cell structure (red circles) and regenerated adipose tissue (red arrow); (**E-e**) N. Caff group showing apoptotic areas (yellow asterisks), disappear of tumor cells identity (red circle) and multiple number of regenerated adipose tissue (red arrows).

Normal mammary fat pad examination (control) revealed normal breast tissue architecture in terms of lobules and ducts. Glands are structured into lobules of complex branching alveolar glands, with a network of ducts terminating in stem-cell enriched structures known as terminal end buds (TEBs), which stimulate additional duct elongation and branching in later embryonic stages. The cellular structure of the ductal-lobular system revealed two layers of cells. These cells have superficially spindle-shaped nuclei, and the inner cuboidal ones. The lumina of the mammary gland alveoli are clean, without cellular debris or secretions (Fig. 9A-a).

The positive control group (PC) indicated Invasive ductal carcinoma with necrosis classified as high grade based on the previously described characteristics. It demonstrated limited necrotic areas (pink homogenous patches) in the center portions of the tumors with a lack of tubular development. Furthermore, the nuclear polymorphism was visible in Fig. 9B-b depicting Plasmacytoid cells, which are cancer cells with an excess of cytoplasm and eccentric nuclei.

Mice treated with *M. oleifera* leaves extract loaded CSNPs exhibited low tumor cell infiltrations, significant necrosis (wide pink regions), and full loss of cellular features, as evidenced by multiple nuclear alterations (pyknosis). Furthermore, it demonstrated low fibrosis formation and tissue degradation. All of these features indicate the *M. oleifera* leaves extract loaded CSNPs' poor selectivity, despite its powerful efficacy against tumor cells (Fig. 9C-c).

In contrast, the Caffeine loaded CsNPs group recovered compared to the positive control group in terms of tumor cell structure (lower number of Plasmacytoid cells). There are also large zones of apoptotic cells and tiny necrotic zones shown. These considerable differences from the *M. oleifera* leaves extract loaded CSNPs group or PC demonstrate Caffeine loaded CsNPs’ selective potency (Fig. 9D-d).

Tumor sections from the *M. oleifera* leaves extract/Caffeine loaded CsNPs group exhibited poorly differentiated cancer cells with low mitotic activity and nuclear polymorphism. It showed a large number of apoptotic cells within the tumor mass in the central areas. According to the categorization grades, this may demonstrate the synergistic impact of *M. oleifera* leaves extract/Caffeine loaded CsNPs in decreasing the carcinoma grade compared to the positive control (Fig. 9E-e).

Furthermore, the growth of adipose tissue in various experimental groups provides a major indicator of therapy, given that the mouse mammary gland is largely composed of adipose tissue that directly connects to ducts without a significant matrix layer. The number of adipose cells recovered was significantly higher in the *M. oleifera* leaves extract/Caffeine loaded CsNPs groups (Fig. 9E-e) than in the other two treatment groups.

### Immunohistochemical examination

#### Qualitative analysis

**Figure 10:**
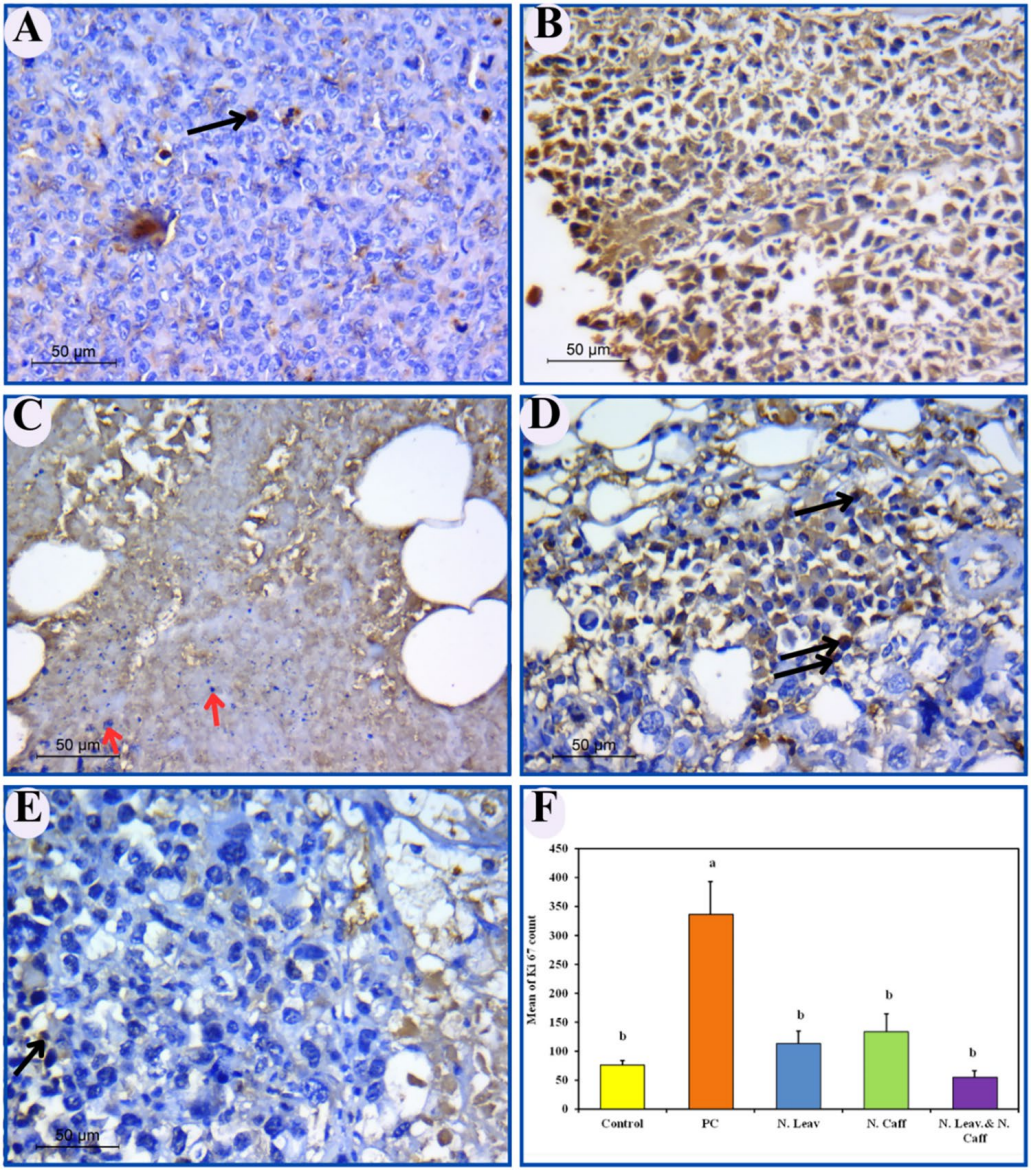
Ki-67 immunohistochemical analysis of mice's breasts in different experimental groups (original magnification × 1000; toluidine blue counterstain). (**A**) Control group showing few Ki-67 positive nuclei (black arrow); (**B**) PC group showing numerous Ki-67 positive cells (black arrows); (**C**) N. Leav. group showing DNA fragments (red arrows); (**D**) N. Caff. group showing some Ki-67 positive nuclei (black arrows); (**E**) N. Leav & N. Caff. group showing rare Ki-67 positive nuclei (black arrow); (**F**) Comparison between different studied groups regarding's ki67 percentage of positive cells (data were described using mean and standard deviation for normally distributed data).

Imunohistochemical examination of ki-67 antibody supported our observation of H & E staining, as low proliferative was found in the Control (Fig. 10A) and *M. oleifera* leaves extract/Caffeine loaded CsNPs (Fig. 10E) groups. On the other hand, The PC group had strong proliferative potential, as revealed by an increase in the quantity of immunological reagent Ki67 with tumor metastasis. (Fig. 10B). The most intriguing finding is that the *M. oleifera* leaves extract loaded CSNPs group has few proliferative nuclei with numerous nuclear fragments, supporting the *M. oleifera* leaves extract loaded CSNPs’ apoptotic ability (Fig. 10C). Similar results were noticed in the caffeine loaded CSNPs’ treated group (Fig. 10D).

#### Quantitative analysis

The percent of positive Ki-67 cells differed significantly between experimental groups, confirming our qualitative assessment supported by the statistical data in Fig. 10F.

### Tumor growth inhibition

**Figure 11:**
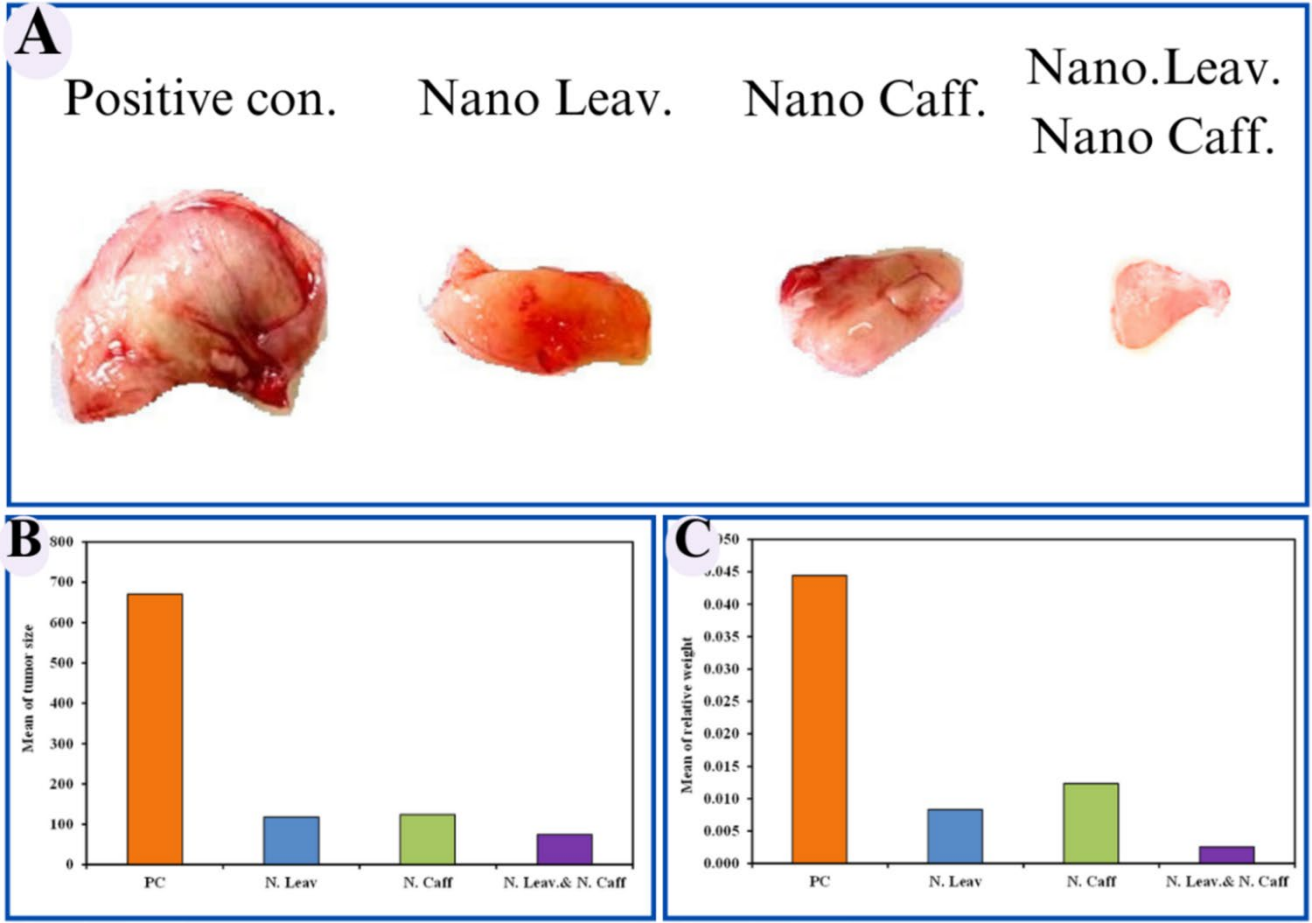
Antitumor effect of different experimental groups 30 days post treatment of Ehrlich ascites mammary tumor in mice showing: (**A**) digital images of excised tumors, (**B**) Percent change in tumor size relative to pretreatment volume and (**C**) Relative tumor weight among the experimental groups.

Morphological evaluation of excised tumors revealed the synergistic effect between *M. oleifera* leaves extract/Caffeine loaded CsNPs by the minimal observed tumor size (Fig. 11A). These results were supported by the statistical data in Fig. 11B, which demonstrates the increase in tumor size percent among the different experimental groups. Additionally, relative tumor weight was evaluated in Fig. 11C, given the same results in significance as relative weight used in studies from toxicological markers^48^

### Molecular investigations

**Figure 12:**
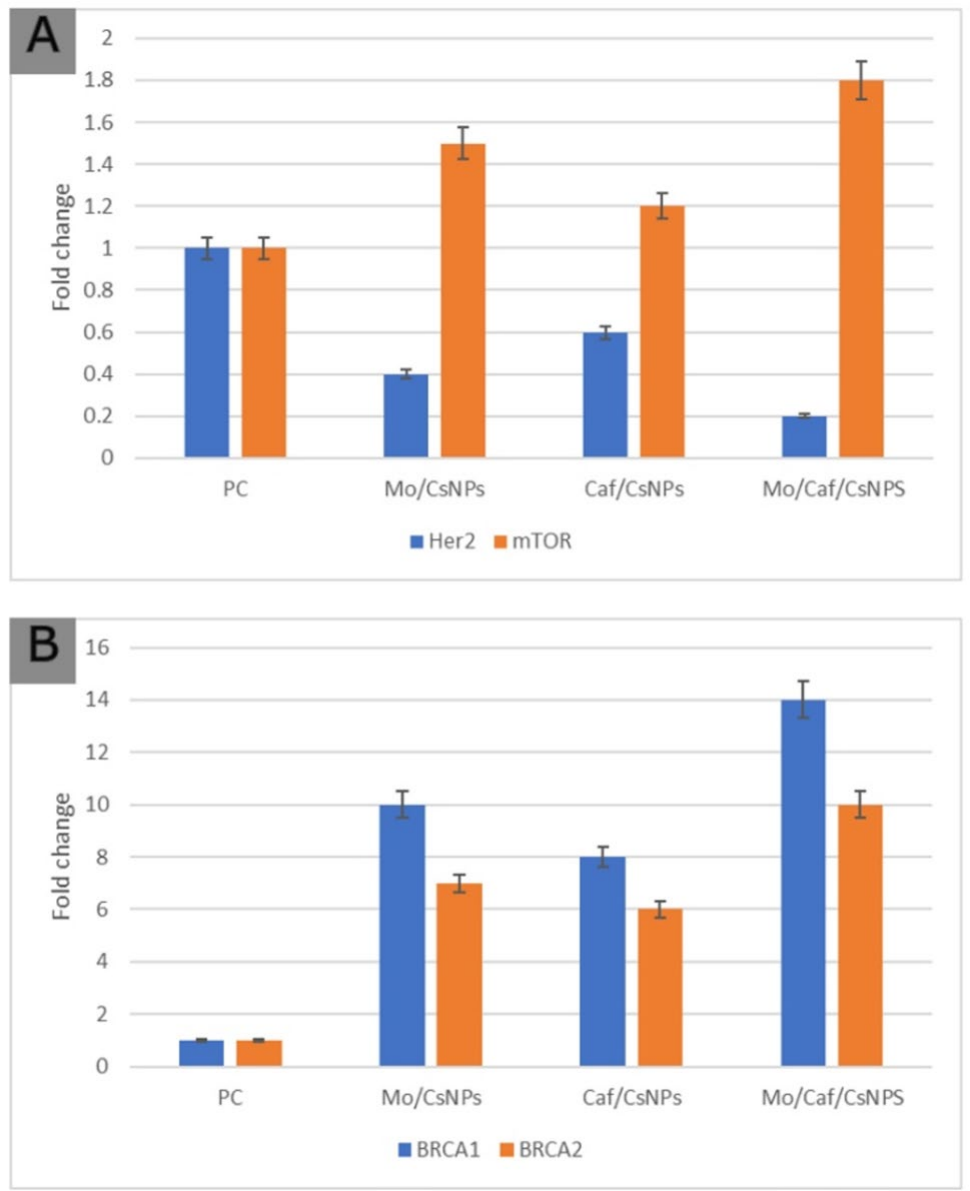
Effect of the formulated nanosystems on levels of Her2, mTOR (**A**), BRCA1 and BRCA2 (**B**). *P < 0.001 from positive control (data were described using mean and standard deviation for normally distributed data).

Recent therapies for BC are based on molecular tumor subtypes that have been labeled by HER2 expression^49^. BC with overexpressed HER2 is more aggressive and correlated with lower prognosis^50^. In the present model of BC (induced with 4T1 cells), Her2 was overexpressed in the positive control group. Treatment with the prepared nanosystems significantly reduced the Her2 expression. Figure 12 shows that BRCA1and BRCA2 expression levels were significantly high (14 and tenfold, respectively) (*p* ≤ 0.05) in the positive control group. Surprisingly, BRCA1 and BRCA2 expression levels were significantly decreased (*p* ≤ 0.05) after Mo/Caf/CsNPs treatment. On the other hand, mTOR expression level showed a 1.8-fold increase in tumor tissue treated with Mo/Caf/CsNPs when compared to the positive control group.

## Discussions

Tamoxifen-stimulated phenotype resistance has been documented in a small percentage of individuals^3^. Moreover, a potent multidirectional antifolate cytotoxic chemotherapeutic medication, pemetrexed (PMT) is used to treat a variety of cancers, including breast cancer. However, the failure to obtain adequate intracellular concentrations within the dosage limits permitted, along with the systemic toxicity and multidrug resistance that arise from raising the PMT dose, limits the therapeutic advantages clinically^51^. Additionally, PMT has poor selectivity and bioavailability. In the present study, we used Moringa leaves extract due to its previously reported antioxidant and anticancer characteristics^52^. Adedapo et al.^53^ study concluded that the plant is relatively safe both for nutritional and medicinal uses. 2000 mg/kg acute toxicity dose showed no mortality rate in Wistar rats and oral sub-acute treatments with 400, 800 and 1600 mg/kg dose showed no significant changes in all the tested organs.

This plant showed anticancer properties by disrupting the signal transduction system that promotes cancer cell proliferation and progression^54^. By using gas chromatography-mass spectrometry (GC–MS), Al-Asmari et al.^55^ discovered that *M. oleifera* includes 12 distinct chemicals, three of which have anticancer characteristics.

On the other hand, caffeine is one of the most widely ingested bioactive substances worldwide. This bioactive substance, which is often present in tea, cocoa, and coffee, belongs to the methylxanthine class^56^. Caffeine can provide a variety of health-benefits, including lowering the risk of diabetes, Alzheimer's, Parkinson's, and cardiovascular diseases, weight loss, boosting liver function, protecting against certain cancers^57,58^. Caffeine-based beverages were considered as natural sources for targeting cancer hallmarks through influencing some molecular pathways, such as apoptosis and DNA damage repair pathways^59^. Hence in the present study rational we aimed to combine and load two naturally known anticancer agents on one nanosystem to synergize their potent effect.

The prepared nanosystem (Mo/Caf/CsNPs) has an average size of 369 nm and zeta potential reached − 36.8 mV while the PDI was 0.321. These results confirmed the stability of the prepared nanoparticles and its suitability for using as anticancer agent. Due to that the abnormal angiogenesis in solid tumours results in a blood vessel that leaks, with inadequate and disorganized endothelial cell junctions^60^. It is anticipated that 100–400 nm diameter nanoparticles would gather at tumour sites via convection and diffusion processes, resulting in the passive increased permeability and retention (EPR) effect^61^. Furthermore, the preservation of the nanocarriers is encouraged by the tumours’ compromised lymphatic drainage^62^. Moreover, Mo/Caf/CsNPs had strong bands that were observed at 1635 (amide C=O stretch), 1566 (amide C=O stretch), and 1240 (OH group) cm^−1^ all which indicated the formation of Moringa/caffeine/chitosan nanoparticles.

In the present study, the prepared nanosystems showed potent anti-cancer effect against MCF-7 cell line with IC_50_ reached 37.81 after 48 h incubation. This effect was further evaluated through in vivo studies. Mo/Caf/CsNPs showed a potent effect on the BC biomarkers namely Ca 15–3, Ca 27–29 and MMP 9. Ca 27–29 antigen is more sensitive than Ca 15–3 and has been approved by the US FDA (United States Food and Drug Administration) for use in detecting the recurrence of BC in patients^63–65^. It is worthy to note that downregulation of TGF-β1/MMP signaling pathway has been an important factor in shifting the cancer progression^66^. Moreover, MMP 9 inhibition has a significant role in the early interruption of the metastatic circuit^67^. In agreement with our results, Rosendahl et al.,^68^ revealed that caffeine has several pharmacological effects. Caffeine could decrease cell proliferation and cause apoptosis in various tumors, including the esophagus, breast, liver, and brain, according to several epidemiological and experimental investigations^68^.

In the present investigations it was noticed that BRCA1 and BRCA 2-genes were overexpressed in 4T1-induced BC. This can be explained by the expected higher proliferation rate in cancer tissues which resulted in genetic instability leading to the urged need for more DNA damage repair. It was revealed by Chandrika et al.^69^ that flavonoids can enhance HER2 positive breast cancer^69^. In another detailed study conducted by Babu et al.^70^ it was announced that the compound ZINC67903192 can be identified as HER2 inhibitor against gastric cancer. Similarly, Wang et al.^71^ reported that mRNA expression levels of BRCA1 and BRCA2 were upregulated in breast and ovarian cancer tissues. Chalabi et al.^72^ explained that BRCA2 overexpression may have a role in BC’s aggressiveness.

*M. oleifera* leaves extract/Caffeine loaded chitosan nanoparticles downregulated the expression of BRCA1 & 2 in tumor tissue. It is well known that BRCA1 interacts with a variety of nuclear proteins, including BRCA2, and hence plays major roles in the cell^72^. The amino terminal ring finger domain of BRCA1 is involved in estrogen receptor signaling repression, DNA repair modulation, and apoptosis. BRCA1’s carboxyl-terminal acidic domain acts as a transcriptional activator when linked to the DNA binding domain. Also, BRCA1 is involved in the control of cell cycle checkpoints and centromeres^73^. Satyananda et al.^74^ speculated that BRCA2 high gene expression in breast cancers is associated with highly proliferative, higher-grade tumors.

## Conclusion

The newly prepared *M. oleifera* leaves extract/Caffeine loaded chitosan nanoparticles showed potent anti-breast cancer effect through downregulating some of the widely known oncogenic genes (Her2, BRCA1 and BRCA2). This was furtherly evident in the histopathological study of the significant difference between the positive control cancer tissue, while the Moringa leaves extract/caffeine loaded chitosan nanoparticles’ treated group showed improved tissue architecture. This proves the novel nanoparticles’ ability to inhibit cancer cells in mice breasts. While further investigations should be done on the Moringa extracts active fractions to assess the exact potential anticancer components. This may pave the way for newly designated chemically synthesized compounds to mimic the observed action and designating of new chemotherapeutics with natural basis.

### Supplementary Information

Supplementary Figure S1.

## Data Availability

All the original data are available upon reasonable request for correspondence authors.
