# Supplementary material for: Newly synthesized chitosan nanoparticles loaded with caffeine/moringa leaf extracts Halt Her2, BRCA1, and BRCA2 expressions
Source: Sci Rep. 2024 Aug 5;14:18118. doi: 10.1038/s41598-024-67599-1 (PMC11300450; doi:10.1038/s41598-024-67599-1)
Supplement: Supplementary file 1 — Supplementary Figure S1. [file 41598_2024_67599_MOESM1_ESM.docx]

**Newly Synthesized Chitosan Nanoparticles Loaded with Caffeine/Moringa Leaf Extracts Halt Her2, BRCA1, and BRCA2 Expressions**

Hanaa Mohammed^1^, Mustafa M. Karhib^2^, Karrar Sabah Jaafar Alfahad^3^, Atef Mohamed Atef^4^, Areej Eskandrani^5^, Amira Abd-elfattah Darwish^6^, Ahmed Abdallah Sary^7^, Bassma H. Elwakil^6,*^ , Basant A. Bakr^8^, Ahmed M Eldrieny^6^

^1^Human Anatomy and Embryology Department, Faculty of medicine, Sohag University, [hanaam415@yahoo.com](mailto:hanaam415@yahoo.com)

^2^Department of Medical Laboratory Techniques, College of Health and Medical Technologies, Al-Mustaqbal University, 51001 Hillah, Babylon, Iraq, [mmk.mk2000@gmail.com](mailto:mmk.mk2000@gmail.com)

^~~3~~^Babylon Education Directorate, Ministry of Education, Iraq, [karraralfihad90@gmail.com](mailto:karraralfihad90@gmail.com)

^4^Faculty of Medical Applied Science, Irbid national university, Irbid, Jordan, a.doweir@inu.edu.jo

^5^College of Science, Taibah University, Madinah 30002, Kingdom of Saudi Arabia, [aeskandrani@gmail.com](mailto:aeskandrani@gmail.com)

^6^Faculty of Applied Health Sciences Technology, Pharos University in Alexandria, Alexandria 21526, Egypt, [bassma.hassan@pua.edu.eg](mailto:bassma.hassan@pua.edu.eg), [amira.mohamed@pua.edu.eg](mailto:amira.mohamed@pua.edu.eg), [ahmed.eldrieny@pua.edu.eg](mailto:ahmed.eldrieny@pua.edu.eg)

^7^Faculty of Physical Therapy, Pharos University in Alexandria, Alexandria 21526, Egypt, [Ahmed.abdallah@pua.edu.eg](mailto:Ahmed.abdallah@pua.edu.eg)

^8^ Faculty of Science, Alexandria University, Alexandria 21321, Egypt, [bassant.kamal.pt@pua.edu.eg](mailto:bassant.kamal.pt@pua.edu.eg)

*Corresponding author email: [Bassma.hassan@pua.edu.eg](mailto:Bassma.hassan@pua.edu.eg)

(a)

(b)

Figure S1. FTIR analysis of the prepared Mo/CsNPs (a), Caf/CsNPs (b)
